# CodonMoE: DNA language models for codon-dependent mRNA prediction

**DOI:** 10.1093/bioinformatics/btag285

**Published:** 2026-05-09

**Authors:** Shiyi Du, Litian Liang, Jiayi Li, Carl Kingsford

**Affiliations:** Ray and Stephanie Lane Computational Biology Department, Carnegie Mellon University, PA 15213, United States; Ray and Stephanie Lane Computational Biology Department, Carnegie Mellon University, PA 15213, United States; Ray and Stephanie Lane Computational Biology Department, Carnegie Mellon University, PA 15213, United States; Ray and Stephanie Lane Computational Biology Department, Carnegie Mellon University, PA 15213, United States

## Abstract

**Motivation:**

Genomic language models (gLMs) face a fundamental efficiency challenge: one must either maintain separate specialized models for each biological modality (DNA and RNA) or develop large multimodal architectures. Both approaches impose significant computational burdens—modality-specific models require redundant infrastructure despite inherent biological connections, while multi-modal architectures demand increased parameter counts and extensive cross-modality pretraining.

**Results:**

To address this limitation, we introduce CodonMoE (Adaptive Mixture of Codon Reformative Experts), a lightweight adapter that transforms DNA language models into effective RNA analyzers without RNA-specific pretraining. Our theoretical analysis establishes CodonMoE as a universal approximator at the codon level, capable of mapping arbitrary functions from codon sequences to codon-dependent RNA properties given sufficient expert capacity. Across four RNA prediction tasks spanning stability, expression, and regulation, DNA models augmented with CodonMoE significantly outperform their unmodified counterparts, with the HyenaDNA+CodonMoE series achieving state-of-the-art results using 80% fewer parameters than specialized RNA models. By maintaining sub-quadratic complexity while achieving superior performance, our approach provides a principled path toward unifying genomic language modeling, leveraging more abundant DNA data and reducing computational overhead while preserving modality-specific performance advantages.

**Availability and implementation:**

Source code for the method and to reproduce the results is available at https://github.com/Kingsford-Group/CodonMoE.

## 1 Introduction

Advancements in Large Language Models (LLMs) are revolutionizing scientific disciplines, with the biomedical sciences experiencing especially profound effects (Jumper *et al.* 2021, Varadi *et al.* 2022). The fundamental goal of Natural Language Processing (NLP) is to comprehend and manipulate sequences of words, a task that bears similarities to one of the central objectives in biology: deciphering the meaning and function encoded in biological sequences (Eraslan *et al.* 2019) and generating novel genomic sequences with desired properties. This parallel has given rise to genomic Language Models (gLMs). GLMs are large-scale language models trained on vast amounts of biological sequence data. These models aim to capture the complex patterns and dependencies within genomic sequences, much like how general LLMs learn the intricacies of human language (Bepler and Berger 2021). By leveraging the power of LLMs and the abundance of genomic data now available, gLMs have the potential to significantly advance our understanding of genomes and reveal how DNA or RNA elements at various scales interact to give rise to biological functions (Zhou *et al.* 2018).

Recent progress in state-space models (SSMs) has addressed the quadratic scaling limitations inherent in self-attention mechanisms, offering efficient alternatives to transformers for gLMs (Ji *et al.* 2021, Benegas *et al.* 2025a, Ratcliff 2024) with subquadratic or linear scaling in sequence length. HyenaDNA (Nguyen *et al.* 2023), built on the Hyena Hierarchy, represents a leap forward in genomic modeling, processing input contexts up to 1 million nucleotides—a 500-fold increase over previous dense attention-based models. This architecture enables single-nucleotide-level analysis across extensive genomic regions, crucial for capturing long-range interactions and subtle genetic variations like SNPs. Caduceus (Schiff *et al.* 2024), leveraging the Mamba-based SSM (Gu and Dao 2024), introduces bi-directionality and reverse complementarity (RC) equivariance, essential properties for DNA sequence analysis. Trained on 131 kb sequences, Caduceus demonstrates superior performance on prediction of long-range effects of variants compared to much larger models. Building upon this framework, PlantCaduceus (Zhai *et al.* 2024) extends these capabilities to plant genomes, showcasing high transferability across species that diverged 160 million years ago and enabling genome-wide deleterious mutation identification without multiple sequence alignment. HELM (Yazdani-Jahromi *et al.* 2025) introduces a hierarchical encoding approach that integrates the biological codon structure of mRNA into language model training, acknowledging that multiple synonymous codons can encode identical amino acids while possessing distinct properties. Evo (Nguyen *et al.* 2024), a hybrid architecture combining Hyena and Transformer elements, pushes the boundaries further with its 7 billion parameter model and 131 kb context length. Evo’s multimodal approach allows it to generalize across DNA, RNA, and protein prediction tasks, while also demonstrating unprecedented capabilities in generating synthetic molecular complexes and coding-rich sequences up to 650 kb in length.

The development of distinct gLMs—encompassing DNA models, RNA models, and multimodal models—introduces a considerable cost burden. This issue becomes increasingly pronounced as the size and complexity of gLMs grow. Moreover, attention-based models, particularly in the context of RNA language modeling, continue to dominate most RNA-specific tasks. Although these models deliver strong performance, their high computational demand remains a substantial challenge. DNA serves as the primary repository of genetic information, while mRNA functions as an intermediary in the expression of this information. Building upon this fundamental concept, DNA-based language models offer a more foundational approach to genomic modeling compared to mRNA-based models. However, despite their great potential, DNA-based models have largely been underused for mRNA tasks.

To address these challenges, we propose a novel approach based on the hypothesis that DNA models can effectively replace RNA models when augmented with RNA-specific control information. We propose an approach called *Adaptive Mixture of Codon Reformative Experts (CodonMoE)* which is a versatile plug-and-play module designed to seamlessly integrate with existing DNA models, transforming them into robust tools for mRNA analyses. We also demonstrate that recent, efficient sub-quadratic DNA-based SSM architectures can be effectively combined with the CodonMoE to yield parameter- and computationally efficient predictions for mRNA tasks. This marks the first approach to bridge the gap between DNA and RNA language models through a universally applicable adapter—CodonMoE. Theoretical proof demonstrates that CodonMoE is a universal approximator of mRNA properties at the codon level. Experimental results further show that CodonMoE significantly enhances various DNA-based backbones by a wide margin. Some of these models achieve performance comparable to or exceeding state-of-the-art (SOTA) mRNA-specific models across several tasks, while also achieving substantial reductions in time complexity and model parameters.

To summarize our contributions:

- We introduce CodonMoE, a plug-and-play adapter that transforms DNA language models into effective RNA analyzers without requiring RNA-specific pretraining.
- We establish CodonMoE as a universal approximator at the codon level, proving it can map arbitrary functions from codon sequences to RNA properties given sufficient expert capacity.
- Our approach maintains low complexity while achieving superior performance, with HyenaDNA+CodonMoE series achieving state-of-the-art results using 80% fewer parameters than specialized RNA models.
- Across RNA prediction tasks spanning stability, expression, and regulation, DNA models augmented with CodonMoE significantly outperform their unmodified counterparts.
- By enabling DNA models to effectively handle RNA tasks, CodonMoE provides a principled path toward unifying genomic language modeling, leveraging more abundant DNA data while reducing the computational overhead of maintaining separate modality-specific models.

### 1.1 Related work

#### 1.1.1 Transformer-based genomic language models

Transformers (Vaswani *et al.* 2017, Devlin *et al.* 2019) have become prevalent in genomics modeling due to their capacity to capture long-range dependencies (Benegas *et al.* 2025b). These models face challenges with extended sequences and nucleotide-level resolution. In DNA modeling, DNABERT (Ji *et al.* 2021) employs k-mer tokenization for tasks like transcription factor binding site prediction. Enformer (Avsec *et al.* 2021) incorporates convolution layers surrounding transformer blocks. Nucleotide Transformer (Dalla-Torre *et al.* 2025) scales to five times DNABERT’s size, while MegaDNA (Shao 2023) extends context windows for longer sequences. GPN-MSA (Benegas *et al.* 2025a, Su *et al.* 2024) uniquely leverages whole-genome alignments across species to enhance DNA modeling. For RNA applications, models such as RNABERT (Akiyama and Sakakibara 2022), BigRNA (Celaj *et al.* 2023), CodonBERT (Li *et al.* 2024), SpliceBERT (Chen *et al.* 2024), NicheFormer (Tejada-Lapuerta *et al.* 2025), and scBERT (Yang *et al.* 2022) address transcriptomic tasks. These RNA-specific models encounter similar limitations as their DNA counterparts regarding sequence length and computational efficiency, motivating the exploration of SSMs as alternatives.

#### 1.1.2 SSM-based genomic language models

State-space models have gained prominence in genomic modeling by offering reduced computational complexity and improved scaling for long-range dependencies. HyenaDNA (Nguyen *et al.* 2023) and Caduceus (Schiff *et al.* 2024) demonstrate effectiveness in sequence modeling with SSM architectures. Evo (Nguyen *et al.* 2024) further extends these capabilities to whole-genome-scale DNA generation, while also demonstrating cross-modality applicability to RNA. HELM (Yazdani-Jahromi *et al.* 2025) introduces a hierarchical encoding approach that explicitly accounts for the codon structure of mRNA, where multiple synonymous codons can encode the same amino acid, highlighting the importance of incorporating biological structure into model training. Despite these advances, SSM architectures face trade-offs in comparing distant sequence positions and capturing bidirectional dependencies compared to attention mechanisms, though these limitations are less critical for codon-level mRNA prediction tasks.

#### 1.1.3 Mixture of experts

MoE architectures enhance model performance through specialized experts focusing on different input aspects. Initially proposed by Jacobs *et al.* (1991) and extended hierarchically by Jordan and Jacobs (1994), MoE was adapted for NLP by Shazeer *et al.* (2017) through sparsely gated activation. Subsequent developments include GShard’s scaling beyond 600 billion parameters (Lepikhin *et al.* 2021), Switch Transformer’s single-expert routing (Fedus *et al.* 2022), GLaM’s energy-efficient scaling (Du *et al.* 2022), and improved sparsity management (Zuo *et al.* 2022). Modern MoE implementations enable efficient scaling while reducing computational requirements through dynamic expert activation.

## 2 Materials and Methods

### 2.1 Overview

We propose CodonMoE, a plug-and-play adapter that transforms pretrained DNA language models into effective mRNA sequence analyzers. The key insight is that DNA and mRNA sequences share codon-level structure, but DNA models lack the fine-grained codon-usage bias information critical for RNA tasks like expression prediction and stability analysis. CodonMoE addresses this gap through a two-stage architecture: (i) a Mixture of Experts (MoE) layer that learns task-specific codon representations from limited RNA training data and (ii) a hierarchical feature extraction module (CodonMoE-pro) that captures multi-scale sequence patterns from the MoE-enriched representations.

**Figure 1:**
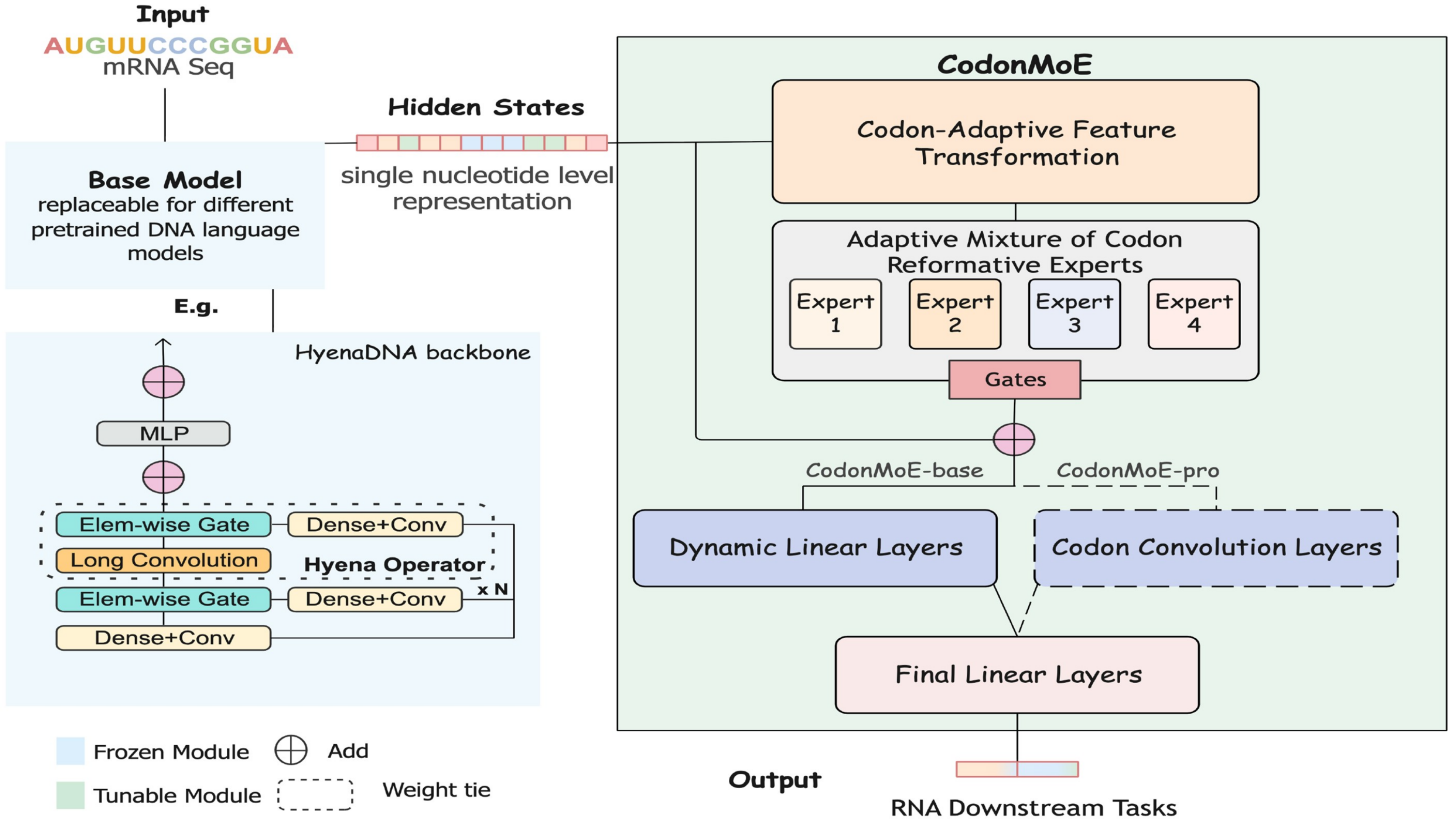
CodonMoE framework. A frozen pretrained DNA model generates nucleotide-level embeddings, which CodonMoE restructures into codon groups. The Mixture of Experts layer learns codon-specific transformations, routing each codon to specialized experts via learned gating weights. The enriched representations are then processed by hierarchical feature extraction modules that capture multi-scale sequence patterns.

Figure 1 illustrates the framework: given a pretrained DNA backbone $h:X\to R^{L\times D}$ (where *L* is sequence length and *D* is embedding dimension), CodonMoE operates on the backbone’s hidden states by (i) reshaping nucleotide-level embeddings into codon-level representations, (ii) applying expert-based transformations that specialize on different codon contexts, (iii) integrating these enriched representations back into the sequence, and (iv) extracting predictive features through multi-scale convolutions. Crucially, only CodonMoE parameters are trained on RNA data—the DNA backbone remains frozen, preserving its pretrained knowledge while adapting to RNA-specific patterns.

### 2.2 CodonMoE architecture

#### 2.2.1 Sample-wise dynamic codon-level representation

CodonMoE processes representations of codons, which are groups of three nucleotides in genetic sequences encoding amino acids. The input to CodonMoE consists of nucleotide representations with dynamic dimensionality, allowing it to accommodate input samples of varying sequence lengths. These inputs are reshaped into codon groups, preserving the structure of the genetic code. The CodonMoE slices this sequence to extract codon-related segments and reshapes them to facilitate further processing.

#### 2.2.2 Adaptive mixture of reformative codon experts

CodonMoE uses Adaptive Mixture of Codon Experts layers, where *k* experts, each specializing in different aspects of the codon data, process these representations. We set K=4, as this provides sufficient capacity to capture major codon usage categories (e.g. high-frequency versus rare codons, GC-rich versus AT-rich) while maintaining parameter efficiency. An ablation over K∈{1,2,4,6,8} (Table 9, available as supplementary data at *Bioinformatics* online) supports this choice. This helps ensure that sparsely occurring, but functionally critical codons receive dedicated capacity. This expert‐driven routing amplifies rare‐codon signals. The transformation is given by:


$$y_{\text{codons}}^{\text{MoE}}=\sum_{k=1}^{K}g_{k}(x)\cdot E_{k}(y_{\;\text{codons}}),$$

where $g_{k}(x)$ is the gating mechanism that determines the contribution of each expert $E_{k}$. This dynamic expert selection allows the MoE to process the codon data in multiple ways, with the gating system controlling which perspective should dominate.

#### 2.2.3 Dynamic reshaping and contextualization

After processing by the experts, the codon-level representations are reshaped to match the original sequence length and structure. The CodonMoE contextualizes this information, enriching it with surrounding data before recombining it with the rest of the input sequence:


$$y_{\text{output}}=y_{\text{reshaped}}+y_{\text{codons}}^{\text{MoE}}.$$

This process ensures that codon-level information is properly embedded and aligned within the original sequence, helping the model recognize both local codon-specific patterns and broader genetic patterns. After residual connection, layer normalization, GELU activation, and dropout are applied to produce the final codon-enriched representations $H_{\text{enrich}}\in R^{B\times L\times D}$, where *B* is batch size, *L* is sequence length, and *D* is embedding dimension. For CodonMoE-base, $H_{\text{enrich}}$ is flattened to a dimension (S−1)×D and projected through a final linear layer Linear((S−1)·D,1) to produce the scalar prediction $\hat{y}\in R^{B\times 1}$. For more detailed specifications of the algorithm, refer to Appendix A.1, available as supplementary data at *Bioinformatics* online.

#### 2.2.4 CodonMoE-pro: hierarchical multi-scale feature extraction

To better capture the diverse sequence patterns in MoE-enriched representations, CodonMoE-pro employs a hierarchical feature extraction module based on convolutional architectures for text classification (Zhang and Wallace 2015). The MoE layer described above operates identically in both CodonMoE-base and CodonMoE-pro; the two variants differ only in their downstream feature extraction. While CodonMoE-base flattens the enriched representations and applies a linear projection, CodonMoE-pro instead employs the convolutional module described above. This module operates directly on the codon-enriched representations $H_{\text{enrich}}$ produced by the preceding MoE and normalization layers. It replaces the final linear projections with a *codon neighborhood convolution*: a narrow sliding-window convolution over adjacent codons. This convolutional layer detects recurring codon pairs or triplets—short motifs known to modulate translation kinetics and mRNA stability. The convolutional filter both suppresses irrelevant patterns and amplifies the most discriminative codon motifs.

We reshape $H_{\text{enrich}}$ to 2D format for convolutional processing, treating the sequence dimension as spatial:


$$H_{\text{enrich}}^{\left(2D\right)}=H_{\text{enrich}}.\text{unsqueeze}(1)\in R^{B\times 1\times L\times D}$$

We then apply multiple parallel 2D convolutions with varying kernel heights K={3,4,5} to extract n-gram features at different scales:


$$C_{k}=\text{ReLU}({\text{Conv}2D}_{k\times D}(H_{\text{enrich}}^{\left(2D\right)}))\in R^{B\times C_{\text{out}}\times (L-k+1)\times 1},$$

where the convolution kernel has height *k* and width *D*, and $C_{\text{out}}$ is the number of output channels (typically 100 per kernel size). Each convolution captures sequence motifs of length *k*: trigram patterns (k=3), 4-gram patterns (k=4), and 5-gram patterns (k=5).

To obtain position-invariant features, we apply max-over-time pooling to each feature map, extracting the most salient feature regardless of position:


$${\tilde{c}}_{k}=\underset{i\in [1,L-k+1]}{\max}C_{k}[:,:,i,0]\in R^{B\times C_{\text{out}}}.$$

The pooled features from all kernel sizes are concatenated:


$$c_{\text{concat}}=[{\tilde{c}}_{3};{\tilde{c}}_{4};{\tilde{c}}_{5}]\in R^{B\times (|K|\cdot C_{\text{out}})}.$$

After dropout regularization, a final linear layer produces the prediction:


$${\hat{y}}_{\text{pro}}=W_{\text{final}}^{⊤}\cdot \text{Dropout}(c_{\;\text{concat}})+b_{\text{final}}.$$

Specifically, with |K|=3 kernel sizes and $C_{\text{out}}=100$ output channels per kernel, the concatenated feature vector $c_{\text{concat}}\in R^{B\times 300}$, and $W_{\text{final}}\in R^{300\times 1}$ projects to the final scalar prediction ${\hat{y}}_{\text{pro}}\in R^{B\times 1}$. This hierarchical design allows CodonMoE-pro to: (i) leverage the codon-aware representations learned by the MoE layer and (ii) extract discriminative multi-scale patterns that are predictive of RNA function. The parallel convolutions capture local motifs at varying granularities, while max-pooling ensures robustness to motif position—properties particularly valuable for genomic sequences where functional elements can occur anywhere along the sequence.

Algorithms 1 and 2 in the appendix, available as supplementary data at *Bioinformatics* online, provide detailed pseudocode for both variants.

### 2.3 Theoretical properties

We establish that CodonMoE retains universal approximation capacity: given sufficient experts *K* and convolutional filters, the architecture can approximate any continuous function $f:C^{m}\to R$ mapping codon sequences to target properties with arbitrary precision (formal statement and proof in Appendix A.2, available as supplementary data at *Bioinformatics* online). This follows from the Universal Approximation Theorem (Hornik *et al.* 1989) applied to the MoE experts and convolutional feature detectors, confirming that our design choices do not fundamentally limit expressiveness.

## 3 Results

### 3.1 Tasks and datasets

We evaluate CodonMoE on 4 RNA tasks, spanning different aspects of RNA biology. The monomeric Red Fluorescent Protein (mRFP) dataset by Nieuwkoop *et al.* (2023) contains 1459 mRFP variants with paired expression levels and sequences, derived from codon-randomized libraries with varying adaptation index biases to analyze sequence variation effects on expression. The task is to predict the expression of these variants from their sequence. The SARS-CoV-2 vaccine degradation dataset by Leppek *et al.* (2022) comprises 2400 samples with measurements of vaccine stability/degradation, providing insights into mRNA vaccine durability factors. The goal is to predict a sequence’s stability. The Tc-riboswitch dataset (Groher *et al.* 2019) contains 355 tetracycline riboswitch dimer sequences positioned upstream of a GFP coding region, with the switching factor measuring differential regulatory effects in the presence versus absence of tetracycline. The goal is to predict the efficacy of the riboswitch. The MLOS dataset (Li *et al.* 2024) includes 164 mRNA candidates encoding influenza hemagglutinin antigen, with fixed untranslated regions and variable coding regions, evaluated for protein expression levels in HeLa cells. The goal is to predict protein levels derived from the given sequence. All datasets were selected to evaluate model performance and match those used in HELM (Yazdani-Jahromi *et al.* 2025) or CodonBERT (Li *et al.* 2024), facilitating direct comparison across key mRNA tasks using consistent 70%/15%/15% train/validation/test splits. All experiments were completed on a single NVIDIA A100 GPU. For more details, see Appendix A.3, available as supplementary data at *Bioinformatics* online.

### 3.2 Model configurations and baselines

Our evaluation spans diverse backbone architectures to thoroughly assess the CodonMoE series (standard CodonMoE and parameter-optimized CodonMoE-pro) as cross-modality adapters. We implement the CodonMoE series across four distinct DNA model paradigms: linear complexity models (GPN-SS (Benegas *et al.* 2025a) Caduceus (Schiff *et al.* 2024)), quadratic complexity attention-based models (GPN-MSA (Benegas *et al.* 2025a)), and sub-quadratic non-attention models (HyenaDNA (Nguyen *et al.* 2023)). This allows us to determine which architectural characteristics are most amenable to cross-modality adaptation and whether efficiency benefits transfer consistently across model families.

For benchmarking, we compare against two categories of baseline methods: (i) state-of-the-art RNA foundation models (CodonBERT (Li *et al.* 2024), RNA-FM (Chen *et al.* 2022, Li *et al.* 2024), SpliceBERT (Chen *et al.* 2024, Yazdani-Jahromi *et al.* 2025), and various transformer/state-space architectures including Transformer XE, Hyena XE, Mamba XE, and Transformer HELM (Vaswani *et al.* 2017, Gu and Dao 2024, Nguyen *et al.* 2023, Yazdani-Jahromi *et al.* 2025)) and (ii) classical feature-engineering approaches (Anand and Jeffrey David 2011, Zhang and Wallace 2015, Li *et al.* 2024) (see Appendix A.5, available as supplementary data at *Bioinformatics* online). This comprehensive comparison framework establishes whether adapting efficient DNA models can outperform both traditional methods and specialized RNA-specific architectures while maintaining computational advantages.

Our analysis examines both absolute performance and relative improvements over baseline models, with special attention to the efficiency-performance trade-offs achieved through cross-modality adaptation using the CodonMoE series. We measure performance using Spearman’s rank correlation, a standard metric for assessing biological property prediction independent of absolute scale. Implementation details, including backbones, hyperparameters, training procedures, and optimization settings are documented in Appendixes A.3 and A.6, available as supplementary data at *Bioinformatics* online.

### 3.3 CodonMoE-pro achieves state-of-the-art performance with fewer parameters

Table 1 demonstrates that HyenaDNA+CodonMoE-pro achieves state-of-the-art results on three out of four RNA tasks while using only 7.5M parameters—91% fewer than CodonBERT (81.7M) and 85% fewer than other leading RNA models (50M). With sub-quadratic complexity O(L log L), our approach outperforms specialized RNA foundation models on vaccine degradation, mRFP expression, and MLOS, demonstrating that properly adapted DNA models can surpass RNA-specific architectures across diverse functionality domains.

**Table 1:** CodonMoE-pro achieves state-of-the-art performance with 85%–91% parameter reduction.^a^

Model	Params	Size	Complexity	Vaccine	mRFP	Tc-ribo.	MLOS
***RNA foundation models***							
RNA-FM	100.00M	□□□	$O(L^{2})$	0.74	0.80	0.58^b^	–
SpliceBERT	20.00M	□□	$O(L^{2})$	0.76	0.60	0.42	–
CodonBERT	81.70M	□□□	$O(L^{2})$	0.77	0.85^c^	0.56	0.54
Transformer XE	50.00M	□□	$O(L^{2})$	0.79^b^	0.82	0.53	0.61^b^
Hyena XE	50.00M	□□	O(L log L)	0.80^c^	0.84^b^	0.52	0.62^c^
Mamba XE	50.00M	□□	O(L)	0.74	0.82	0.52	0.62^c^
Transformer HELM	50.00M	□□	$O(L^{2})$	0.79^b^	0.85^c^	**0.62**	0.59
***DNA Foundation Models Enhanced with CodonMoE-pro***							
HyenaDNA + CodonMoE-pro (ours)	7.50M	□	O(L log L)	**0.84**	**0.88**	0.60^c^	**0.63**

a Comparison of computational complexity and Spearman’s rank correlation across RNA foundation models and DNA models enhanced with CodonMoE-pro. **Params** column shows total model parameters. **Size:**  □  < 10M, □□ = 20–80M, □□□  > 80M; **Performance: Bold** = best,

b third-best,

c second-best; Missing values indicate model unable to process due to sequence length limitations; HyenaDNA+CodonMoE-pro achieves SOTA with only 9% of CodonBERT’s parameters.

### 3.4 Architectural design progression: from global averaging to local context modeling

To understand the contribution of each architectural component, Table 2 presents a systematic progression from simple codon averaging (CodonMean) through expert-based routing (CodonMoE-base) to our complete design with neighborhood convolution (CodonMoE-pro). CodonMean is a minimal baseline adapter that computes the arithmetic mean of the three nucleotide embeddings forming each codon. By aggregating global codon usage bias, CodonMean provides a crude correction to DNA backbones’ neglect of synonymous codon effects. On both mRFP expression and vaccine degradation tasks, CodonMean delivers a solid lift in Spearman’s ρ (Table 2), demonstrating that even a simple adapter can yield gains. However, its uniform averaging inherently masks low‐frequency “hotspot” codons and fails to differentiate context‐specific patterns, which caps its maximum benefit.

**Table 2:** Design space exploration: progressive enhancement from global averaging to local context modeling. ^a^

	Vaccine degradation		mRFP expression	
	GPN-MSA	HyenaDNA	GPN-MSA	HyenaDNA
Base model	0.57	0.70	0.33	0.44
CodonMean	0.73	0.79	0.74	0.77
CodonMoE-base	0.77	0.81	0.79	0.84
CodonMoE-pro	**0.82**	**0.84**	**0.81**	**0.88**

a Each architectural component contributes to improved performance (Spearman’s rank correlation). Bold values indicate the highest performance in each column.

Each enhancement brings consistent improvements, with CodonMoE-pro achieving the highest performance through its dual capability: specialized per-codon processing via mixture-of-experts and local interaction detection via neighborhood convolution. The progressive gains—particularly the jump from CodonMean to CodonMoE-base and from CodonMoE-base to CodonMoE-pro—demonstrate that both codon-level specialization and local context modeling are essential for capturing translation dynamics.

### 3.5 CodonMoE-pro generalizes across DNA architectures

Table 3 demonstrates that CodonMoE-pro consistently enhances diverse DNA backbones. The gains are particularly pronounced for models with initially weaker RNA task performance: architectures exhibiting lower baseline correlations show substantially larger absolute improvements, with some configurations demonstrating relative gains exceeding 100%. The consistent improvements across architectures with different computational complexities—from sub-quadratic to quadratic scaling—demonstrate the adapter’s versatility and robustness across diverse architectural paradigms.

**Table 3:** CodonMoE-pro generalizes across diverse DNA architectures.^a^

DNA backbone	Complexity	Vaccine degradation			mRFP expression		
		Base	+CodonMoE-pro	Δ	Base	+CodonMoE-pro	Δ
**GPN-MSA**	$O(L^{2})$	**0.55**	**0.82**	**+0.27**	**0.33**	**0.81**	**+0.48**
**HyenaDNA**	O(L log L)	0.69	**0.84**	+0.15	0.44	**0.88**	+0.44

a Comparison shows consistent improvements with substantial gains for initially weaker models. Bold values indicate state-of-the-art results. Despite using DNA-pretrained backbones, CodonMoE-pro achieves superior performance compared to specialized RNA models (see Table 1). The substantial Δ values for initially weaker models demonstrate that CodonMoE-pro bridges fundamental DNA-RNA representational gaps.

### 3.6 Backbone comparison: consistent enhancement patterns

Table 4 provides a comprehensive view of both CodonMoE-base and CodonMoE-pro across multiple DNA backbones. Models with initially poorer RNA task performance show the most improvements when augmented with either adapter variant, with CodonMoE-pro consistently achieving 2%–5% higher correlation than CodonMoE-base. The results show that configurations using 7–13M total parameters (e.g. HyenaDNA+CodonMoE-pro at 7.5M and HyenaDNA+CodonMoE-base at 12.7M) achieve high performance, suggesting that strategic parameter allocation dedicating capacity to codon-specific experts and local context convolution matters more than raw parameter count. For additional cross-backbone comparisons, see Appendix A.5, available as supplementary data at *Bioinformatics* online.

**Table 4:** Comparison of DNA backbones with CodonMoE variants.^a^

Method	Compl.	# Params	Vaccine	mRFP
GPN-SS	O(L)	65.6M	0.60	0.56
+CodonMoE-base	O(L)	78.2M	0.74	0.82
GPN-MSA	$O(L^{2})$	85.7M	0.55	0.33
+CodonMoE-base	$O(L^{2})$	161.9M	0.77	0.79
+CodonMoE-pro	$O(L^{2})$	115.0M	0.82	0.81
Caduceus	O(L)	7.7M	0.56	0.49
+CodonMoE-base	O(L)	48.1M	0.80	0.80
HyenaDNA	O(L log L)	4.1M	0.69	0.44
+CodonMoE-base	O(L log L)	12.7M	0.81	0.84
+CodonMoE-pro	O(L log L)	7.5M	**0.84**	**0.88**

a Both adapter types consistently enhance performance, with CodonMoE-pro achieving superior results through local context modeling. All DNA backbones evaluated on identical train/validation/test splits (70%/15%/15%). **Bold** denotes state-of-the-art performance. CodonMoE-pro consistently outperforms CodonMoE-base while often using fewer parameters (compare HyenaDNA: 7.5 versus 12.7M).

### 3.7 Architectural benefits beyond parameter count

To isolate the contribution of our architectural design from parameter count effects, Table 5 compares three HyenaDNA configurations with controlled parameters: a dense baseline (equivalent parameters to CodonMoE-pro), CodonMoE-base, and CodonMoE-pro. To serve as a rigorous baseline, the dense model replaces the CodonMoE module with a standard multi-layer perceptron (MLP) adapter. This dense MLP is trained to project the DNA embeddings for the downstream RNA tasks while strictly maintaining an equivalent number of trainable parameters and identical training hyperparameters to our proposed models.

**Table 5:** Architectural design versus parameter count: CodonMoE-pro outperforms parameter-matched dense baseline. ^a^

Model	Vaccine	mRFP	Tc-ribo.	MLOS
+Dense baseline	0.80	0.82	0.50	0.55
+CodonMoE-base	0.81	0.84	0.55	0.56
+CodonMoE-pro	**0.84**	**0.88**	**0.60**	**0.63**

a Comparison demonstrates that performance gains derive from structural innovations rather than capacity increase (Spearman’s rank correlation). Dense baseline uses equivalent parameters to CodonMoE-pro with standard fully connected layers. The base model is HyenaD. Bold values indicate the highest performance in each column.

Despite having similar or fewer parameters than the dense baseline, CodonMoE-pro achieves substantial performance gains across all four tasks, with particular improvements on MLOS and vaccine degradation. The consistent superiority of CodonMoE-pro over both the dense baseline and CodonMoE-base indicates three architectural advantages: (i) mixture-of-experts provides specialized pathways for functionally critical codons that dense layers cannot replicate, (ii) codon neighborhood convolution captures local mRNA patterns useful for prediction, and (iii) sparse expert activation inherently regularizes training, reducing overfitting compared to fully connected architectures.

These ablations reveal distinct task-specific architectural requirements: vaccine degradation and MLOS benefit most from local context modeling (CodonMoE-pro’s convolutional component), while mRFP and Tc-riboswitch show strong gains from expert specialization alone (CodonMoE-base). This pattern aligns with biological mechanisms: vaccine stability depends on local RNA structure motifs detectable through convolution, whereas expression levels are influenced by both local and distributed codon usage patterns captured by the mixture-of-experts. For additional experiments, refer to Appendix A.4, available as supplementary data at *Bioinformatics* online.

### 3.8 Raw DNA embeddings lack mRNA-specific guidance across diverse tasks

To systematically evaluate how much mRNA-relevant information DNA models capture without adaptation, we froze the pretrained embeddings of two representative backbones—GPN-MSA (attention-based) and HyenaDNA (non-attention-based)—and trained standard regressors (MLP and XGBoost) across all four RNA tasks (Table 6). The results reveal a consistent performance ceiling: even with powerful tree-based regressors, raw DNA embeddings achieve only modest correlations, substantially trailing both RNA-specialized models and CodonMoE-enhanced variants.

**Table 6:** Raw DNA embeddings require mRNA-specific adaptation across diverse tasks.^a^

	Vaccine		mRFP		Tc-ribo.		MLOS	
Method	GPN	Hyena	GPN	Hyena	GPN	Hyena	GPN	Hyena
MLP	0.57	0.70	0.33	0.44	0.37	0.41	–	0.57
XGBoost	0.75	0.71	0.48	0.51	0.42	0.18	–	0.59

a Frozen backbone embeddings with standard regressors (MLP and XGBoost) in Spearman’s rank correlation. Missing values indicate model unable to process due to sequence length limitations.

Task-specific patterns emerge from the data. Tree-based regressors perform reasonably well on vaccine degradation with attention-based backbones, suggesting DNA pretraining captures some sequence stability patterns. However, performance on regulatory element prediction (Tc-riboswitch) drops dramatically, indicating that such functions require specialized modeling beyond what DNA embeddings provide. Non-attention backbones show more consistent but moderate performance across tasks, yet still fall short of adapted models. The substantial gaps between raw embeddings and CodonMoE-enhanced models, with improvements ranging from minimal to several-fold increases in correlation, demonstrate that architectural adaptation rather than just sophisticated downstream processing is essential for effective DNA-to-RNA transfer. See Appendix, available as supplementary data at *Bioinformatics* online, for additional discussion.

## 4 Discussion

CodonMoE provides a parameter-efficient approach for adapting pretrained DNA language models to codon-dependent RNA prediction tasks. Across several mRNA datasets evaluated, the framework demonstrates consistent improvements when integrated with different DNA backbone architectures. The key practical advantage is the ability to leverage existing pretrained DNA models for RNA applications without requiring RNA-specific pretraining.

Our results suggest that this adapter-based approach may offer a computationally efficient alternative to maintaining separate specialized models: HyenaDNA+CodonMoE-pro achieves state-of-the-art or competitive performance on three of four tasks while using substantially fewer parameters (7.5M) than specialized RNA models (50–100M). Our current evaluation focuses on mRNA prediction tasks where codon-level features are functionally relevant, spanning expression prediction, stability assessment, and regulatory element efficacy. Whether this approach generalizes to broader RNA tasks and species beyond those tested here remains to be explored in future work.

### 4.1 Task-specific performance patterns

We observe notable variation in performance gains across tasks. Linear complexity models show strong results on vaccine stability prediction but more modest improvements on mRFP expression tasks. One possible interpretation is that vaccine stability may depend more on global sequence patterns that SSMs capture effectively, while expression prediction may require finer-grained local features. However, these biological interpretations remain hypothetical and would require targeted experimental validation to confirm the underlying mechanisms.

### 4.2 Backbone architecture considerations

Our results reveal that SSM-based backbones (e.g. HyenaDNA) benefit more from CodonMoE than Transformer-based alternatives (e.g. GPN-MSA), both in absolute performance and parameter efficiency. We attribute this to three factors. First, HyenaDNA was pretrained on the human reference genome at single-nucleotide resolution over 1M-token contexts, providing richer local sequence representations that serve as a stronger foundation for codon-level adaptation. Second, SSM architectures capture long-range dependencies through their state-space formulation, which complements CodonMoE’s local codon-level processing; in contrast, Transformer attention mechanisms may already encode some local patterns that partially overlap with CodonMoE’s function, yielding smaller marginal gains. Third, sub-quadratic models allow more efficient parameter allocation; the full HyenaDNA+CodonMoE-pro system (7.5M parameters) achieves superior performance compared to quadratic-complexity alternatives with 10–20× more parameters. These observations suggest that CodonMoE is most effective when paired with backbones that (i) are pretrained on large-scale genomic data with nucleotide-level resolution, (ii) natively capture long-range dependencies, and (iii) operate with sub-quadratic complexity, leaving a parameter budget for the adapter.

### 4.3 Parameter efficiency observations

CodonMoE-pro variants with 7–13M total parameters often match or exceed the performance of larger configurations. This suggests that strategic parameter allocation to codon-specific processing may be more important than raw parameter count, though the exact relationship requires further systematic investigation across more model sizes and architectures. Our structured design-space exploration helps explain why more expressive adapters—beyond trivial parameter increases—are architecturally aligned with the underlying patterns in mRNA. The observed pattern could indicate diminishing returns beyond a certain adapter capacity. Future work should systematically explore the parameter-performance trade-off across a broader range of model sizes, architectures, and adapter configurations to establish principled guidelines for optimal parameterization in genomic sequence adapters.

### 4.4 Relationship to RNA language models

CodonMoE addresses a challenge complementary to RNA language model development: rather than building increasingly large RNA-specific architectures, it enables the reuse of pretrained DNA models for RNA tasks through lightweight adaptation. For RNA language models that are already pretrained on RNA data (e.g. RNA-FM, CodonBERT), CodonMoE is not the intended solution, as these models have already internalized codon-level and translation-related features during pretraining. Instead, our key finding is that DNA+CodonMoE (7.5M parameters) can match or exceed specialized RNA models with 10–13× more parameters, suggesting that separate large-scale RNA pretraining may not always be necessary. This offers a more resource-efficient path, particularly as DNA pretraining data are more abundant and efficient DNA architectures continue to advance rapidly. An interesting future direction would be applying MoE-based sparse activation principles within RNA model architectures to improve their own parameter efficiency, though this constitutes a separate research question from cross-modal adaptation.

### 4.5 Interpretability of learned expert specializations

To provide insight into the biological mechanisms captured by CodonMoE, we conducted post-hoc analyses of the expert gating weights and convolutional filter activations on the MLOS dataset. The four experts learn distinct, non-redundant specializations aligned with known codon biology (see Section A.7, available as supplementary data at *Bioinformatics* online, for detailed analyses).

### 4.6 Limitations and scope

CodonMoE shows weaker performance on the Tc-riboswitch task compared to other benchmarks. Since riboswitches are non-coding regulatory elements whose function depends on RNA secondary structure rather than translation-related codon patterns, this limitation aligns with the adapter’s design focus on codon-level features. The approach appears most effective for translation-related prediction tasks (expression, stability) where codon usage and local sequence context are functionally relevant. For tasks heavily dependent on RNA structural features independent of codon biology, alternative or complementary approaches may be needed. This limitation extends to other structure-dependent tasks such as RNA tertiary structure prediction, which relies on nucleotide-level spatial interactions and long-range 3D contacts that are not captured by codon-level representations. More broadly, CodonMoE is most effective for downstream tasks where codon-level information is functionally relevant, such as expression prediction, stability assessment, and translation regulation, while the scope of applicable tasks also depends on the capabilities of the underlying DNA backbone. Future work should explore whether incorporating explicit structural information could address this limitation while maintaining the adapter’s efficiency advantages.

## Supplementary Material

btag285_Supplementary_Data

## Data Availability

The datasets used in this study are publicly available from the sources cited in the manuscript and Supplementary Material.
