# Supplementary material for: CodonMoE: DNA language models for codon-dependent mRNA prediction
Source: Bioinformatics. 2026 May 9;42(6):btag285. doi: 10.1093/bioinformatics/btag285 (PMC13224961; doi:10.1093/bioinformatics/btag285)
Supplement: btag285_Supplementary_Data [file btag285_supplementary_data.pdf]

A. Technical appendices and supplementary material

Supplementary material for "S. Du, L. Liang, J. Li, and C. Kingsford. CodonMoE: DNA Language Models for Codon-Dependent mRNA Prediction".

A.1. Algorithm pseudocode and details

The proposed CodonMoE and CodonMoE-pro (Algorithms 1 and 2), efficiently analyze mRNA sequences by leveraging a novel MoE model tailored for codon-level feature extraction. These methods are designed to operate on the hidden representations produced by a base model trained on DNA sequences, improving mRNA sequence analysis through a codon-level adapter. Below, we outline the core components of these algorithms.

Input and hidden representation

The algorithm takes as input hidden states

$$H \in \mathbb{R}^{\text{batch\_size} \times \text{seq\_len} \times d_{\text{model}}},$$

where H is the latent representation generated by a base model trained on nucleotide-level tokenized DNA sequences. These hidden states encapsulate nucleotide-level patterns in the DNA sequence but lack the explicit codon-level representation required for understanding mRNA translation and regulation. CodonMoE restructures these hidden states to focus on codon-level features for better-adapting DNA models for mRNA analysis.

Codon aggregation and reshaping

mRNA sequences consist of codons, which are triplets of nucleotides fundamental to protein synthesis. The hidden states H are reshaped into groups of three consecutive hidden vectors to form codon-level representations. Specifically, the tensor is reshaped into $[B, S/3, 3d]$, where each codon consists of three concatenated hidden vectors. This step captures interactions between nucleotides within each codon.

Mixture of Experts (MoE) for codon-level feature learning

At the core of the CodonMoE is a MoE mechanism that selects from multiple expert networks to process codon-level representations dynamically. Each codon is processed by num_experts linear sub-networks (experts), where each expert specializes in extracting different semantic aspects of the codon. The outputs of these experts are weighted by a softmax gating mechanism, conditioned on the codon input. This ensures the CodonMoE mechanism is highly adaptable to varying contexts within RNA sequences.

Codon-level expansion and integration

After extracting codon-level features from the MoE, these features are expanded to match the original sequence length by repeating the codon features three times, once for each nucleotide in the codon. This expanded representation is reshaped back to $[B, S-1, d]$ and added element-wise to the original hidden states. The result is an enhanced representation that incorporates both nucleotide-level and codon-level information, improving the model's ability to capture local patterns and broader codon interactions.

Regularization and transformation

To ensure robust learning and prevent overfitting, the algorithm applies a series of regularization and transformation steps:

- **Layer normalization**: Ensures stability during training by normalizing the feature map.
- **GELU activation**: Introduces non-linearity to enhance the model's ability to learn complex relationships between codon sequences and biological function.
- **Dropout**: Prevents overfitting by randomly dropping units during training, particularly useful for high-dimensional biological data.

The final feature map is then flattened and passed through a linear transformation, producing a compact feature vector Y that can be used for downstream tasks, such as mRNA classification or regression.

CodonMoE-pro: hierarchical multi-scale feature extraction

CodonMoE-pro replaces the final feedforward layers with a hierarchical feature extraction module from convolutional architectures for text classification (Zhang and Wallace, 2015). This module employs *codon neighborhood convolution*: a sliding-window convolution over adjacent codons that detects recurring codon pairs or triplets—short motifs known to modulate translation kinetics and mRNA stability. The module applies multiple parallel 2D convolutions with kernel heights $\mathcal{K} = \{3, 4, 5\}$ to extract n-gram features at different scales, capturing trigram, 4-gram, and 5-gram sequence patterns. Max-over-time pooling ensures position-invariant feature extraction. The pooled features from all kernel sizes are concatenated and passed through a final linear layer to produce the prediction. This design leverages codon-aware MoE representations while extracting discriminative multi-scale patterns predictive of RNA function.

A.2. Proof of universality

Theorem A.1. *Let $\mathcal{C} = \{A, C, G, T\}^3$ be the codon space, and let $\mathcal{F} = \{f : \mathcal{C}^n \to \mathbb{R}\}$ be the class of target functions. Consider a pretrained backbone model $h : \mathcal{X} \to \mathbb{R}^{L \times D}$, where $\mathcal{X} = \{A, C, G, T\}^*$, and an adapter CodonMoE $g : \mathbb{R}^{L \times D} \to \mathbb{R}$ structured as a dense MoE with K experts. Assume the following conditions hold:*

Algorithm 1 CodonMoE for mRNA Sequence Analysis

Require: Hidden states $H \in \mathbb{R}^{\text{batch\_size} \times \text{seq\_len} \times d_{\text{model}}}$
Ensure: Feature vector Y

```
Hyperparameters: num_experts ← 4, dropout_rate ← 0.1

function MixtureOfExperts(X)
    for i ← 1 to num_experts do
        expert_i ← Sequential(Linear(3d, 3d), GELU, Linear(3d, d))
        outputs[i] ← expert_i(X)
    end for
    gate ← Softmax(Linear(3d, num_experts)(X))
    return Σ_{i=1}^{num_experts} outputs[i] ⊙ gate[:, :, i]
end function

function MoEAdapter(H, finalLayer)
    (B, S, d) ← shape(H)
    Y ← H[:, 0 : S − 1, :]
    codons ← Reshape(Y, [B, ⌊(S − 1)/3⌋, 3d])
    moe ← MixtureOfExperts(codons)
    expanded ← Repeat(moe, 3, dim = 1)
    expanded ← Reshape(expanded, [B, S − 1, d])
    Y ← Y + expanded
    Y ← Dropout(GELU(LayerNorm(Y)), dropout_rate)
    Y ← Linear((S − 1)d, d)(Flatten(Y))
    Y ← Dropout(GELU(LayerNorm(Y)), dropout_rate)
    return finalLayer(Y)
end function

function CodonMoE(sequence)
    tokens ← Tokenize(sequence)
    hidden ← BaseModel(tokens)
    return MoEAdapter(hidden, Linear(d, 1))
end function
```

Algorithm 2 CodonMoE-pro for mRNA Sequence Analysis

Require: Hidden states $H \in \mathbb{R}^{\text{batch\_size} \times \text{seq\_len} \times d_{\text{model}}}$
Ensure: Prediction value $\hat{y}_{\text{pro}} \in \mathbb{R}^{B \times 1}$

```
Convolution parameters: K ← {3, 4, 5}, C_out ← 100

function CodonNeighborhoodConv(Y ∈ R^{B×(S−1)×d})
    Y^(2D) ← Reshape(Y, [B, 1, S − 1, d])
    for k ∈ K do
        C_k ← ReLU(Conv2D(Y^(2D); kernel = (k, d), out = C_out))
        c̃_k ← MaxPool(C_k, dim = 2)                          ▷ Max-over-time
    end for
    c ← Concat([c̃_3, c̃_4, c̃_5])
    return W_out^⊤ Dropout(c, dropout_rate) + b_out
end function

function CodonMoE-pro(sequence)
    tokens ← Tokenize(sequence)
    hidden ← BaseModel(tokens)
    return MoEAdapter(hidden, CodonNeighborhoodConv)
end function
```

1. ***Expert capacity:*** *Each expert $E_k : \mathbb{R}^D \to \mathbb{R}^{D'}$ within the MoE is a neural network capable of uniformly approximating any continuous function on compact subsets of $\mathbb{R}^D$.*
2. ***Gating mechanism:*** *The gating network $G : \mathbb{R}^D \to \Delta^K$ (where Δ^K is the K-simplex) assigns non-negative weights $g_k(z_i)$ to each expert based on the input $z_i \in \mathbb{R}^D$, satisfying $\sum_{k=1}^{K} g_k(z_i) = 1$.*
3. ***Embedding representation:*** *Each DNA sequence $x \in \mathcal{X}$ is partitioned into codons $(c_1, c_2, \ldots, c_n)$, and the backbone model generates embeddings $h(x) \in \mathbb{R}^{L \times D}$, where $L = 3n$ (assuming each codon is represented by three consecutive embeddings).*

Then, for any function $f \in \mathcal{F}$ and for any $\epsilon > 0$, there exists a number of experts K and corresponding parameters for the CodonMoE such that, for all $x \in \mathcal{C}^n$, the approximation error satisfies

$$\left| f(c_1, c_2, \ldots, c_n) - g\left(\sum_{i=1}^{n} \sum_{k=1}^{K} g_k(z_i) \cdot E_k(z_i) \right) \right| < \epsilon,$$

where $z_i = [h(c_i)] \in \mathbb{R}^D$ is codon c_i represented by averaging three nucleotide embeddings.

Proof We aim to show that the CodonMoE, functioning as an adapter to the pretrained DNA backbone h, is a universal approximator for any function $f \in \mathcal{F}$, where $\mathcal{F}$ is the class of continuous functions mapping codon sequences to target properties.

Let $x \in \mathcal{X}$ be a sequence partitioned into n codons:

$$x = (c_1, c_2, \ldots, c_n), \quad c_i \in \mathcal{C}.$$

The backbone model $h : \mathcal{X} \to \mathbb{R}^{L \times D}$ with $L = 3n$ generates embeddings:

$$h(x) = [e_1, e_2, \ldots, e_L]^\top \in \mathbb{R}^{L \times D}.$$

Each codon c_i is represented by averaging three nucleotide embeddings:

$$z_i = \frac{e_{3i-2} + e_{3i-1} + e_{3i}}{3} \in \mathbb{R}^D.$$

The CodonMoE applies a Mixture of Experts model to each z_i:

$$f_{\text{MoE}}(z_i) = \sum_{k=1}^{K} g_k(z_i) \cdot E_k(z_i),$$

where:

$$g_k(z_i) = \frac{\exp(\phi_k(z_i))}{\sum_{j=1}^{K} \exp(\phi_j(z_i))},$$

with gating functions $\phi_k : \mathbb{R}^D \to \mathbb{R}$, and expert networks $E_k : \mathbb{R}^D \to \mathbb{R}^m$.

By the Universal Approximation Theorem (Hornik et al., 1989), for each f_k and any $\epsilon > 0$, there exists E_k such that:

$$\|E_k(z_i) - f_k(z_i)\| < \frac{\epsilon}{Kn},$$

where $f_k \in C(\mathbb{R}^D, \mathbb{R}^m)$.

Define the overall network function:

$$F(x) = \sum_{i=1}^{n} f_{\text{MoE}}(z_i) = \sum_{i=1}^{n} \sum_{k=1}^{K} g_k(z_i) \cdot E_k(z_i).$$

For the target function $f \in \mathcal{F}$:

$$f(x) = \sum_{i=1}^{n} f_i(z_i), \quad f_i \in C(\mathbb{R}^D, \mathbb{R}^m).$$

Then, the approximation error is:

$$\|F(x) - f(x)\| = \left\| \sum_{i=1}^{n} \sum_{k=1}^{K} g_k(z_i) \cdot E_k(z_i) - \sum_{i=1}^{n} f_i(z_i) \right\|.$$

Given that $\sum_{k=1}^{K} g_k(z_i) = 1$ and $g_k(z_i) \geq 0$, we have:

$$\begin{aligned} \|F(x) - f(x)\| &\leq \sum_{i=1}^{n} \sum_{k=1}^{K} g_k(z_i) \|E_k(z_i) - f_i(z_i)\| \\ &< \sum_{i=1}^{n} \sum_{k=1}^{K} g_k(z_i) \frac{\epsilon}{Kn} = \frac{\epsilon}{K}. \end{aligned}$$

The backbone model h ensures that embeddings z_i capture essential genetic information:

$$h : \mathcal{X} \to \mathbb{R}^{L \times D}, \quad z_i = \mathcal{P}(h(x)),$$

where $\mathcal{P}$ denotes the partitioning into codon embeddings via averaging.

Table 7 Summary of experimental settings for different datasets in this work.

Backbone	Model	Backbone Name	Regressor	Learning Rate	Epochs
Caduceus	Caduceus	caduceus-ps_seqlen-1k_d_model-256_n_layer-4_lr-8e-3	mlp	-	-
Caduceus	Caduceus	caduceus-ps_seqlen-1k_d_model-256_n_layer-4_lr-8e-3	xgboost	-	-
Caduceus	Caduceus+CodonMean	caduceus-ps_seqlen-1k_d_model-256_n_layer-4_lr-8e-3	-	0.0005	100
Caduceus	Caduceus+CodonMoE	caduceus-ps_seqlen-1k_d_model-256_n_layer-4_lr-8e-3	-	0.0005	100
HyenaDNA	HyenaDNA	hyenadna-small-32k-seqlen	mlp	-	-
HyenaDNA	HyenaDNA	hyenadna-small-32k-seqlen	xgboost	-	-
HyenaDNA	HyenaDNA+CodonMean	hyenadna-small-32k-seqlen	-	0.0005	100
HyenaDNA	HyenaDNA+CodonMoE	hyenadna-small-32k-seqlen	-	0.0001(0.001)	100

Combining the above, for any $f \in \mathcal{F}$ and $\epsilon > 0$, there exists a CodonMoE network such that:

$$\|F(x) - f(x)\| < \epsilon.$$

Thus, the CodonMoE integrated with the pretrained backbone h satisfies:

$$F = \sum_{i=1}^{n} \sum_{k=1}^{K} g_k(z_i) \cdot E_k(z_i) \approx f(x), \quad \forall f \in \mathcal{F}.$$

Therefore, the CodonMoE module, when combined with the pretrained backbone model h, serves as a universal approximator for any continuous function mapping codon sequences to target properties within the class $\mathcal{F}$. □

A.3. Additional experimental details

Experimental settings

Table 7 outlines the key components and hyperparameters used for different backbone models, highlighting the settings in regressor types and training parameters such as learning rates and the number of epochs. Specifically, it details the setup for the mRFP expression dataset, using Caduceus and HyenaDNA as primary backbones with variations such as Caduceus+CodonMean and Caduceus+CodonMoE, indicating different CodonMoE variations within the same framework. Specific configurations such as the backbone sequence length, model dimensions, number of layers, and learning rates are listed, with pure backbone models integrating machine learning regressors like MLP and XGBoost. It also outlines settings for the SARS-CoV-2 vaccine degradation, MLOS and Tc-riboswitch dataset with similar backbone models but slightly adjusted parameters, such as a different sequence length for the HyenaDNA models. This table showcases the learning rates and epochs where applicable, providing a comprehensive view of how each model is tuned for its respective task.

Dataset details

For the mRFP expression dataset, Nieuwkoop et al. (2023) constructed low (CAI_L), medium (CAI_M), and high (CAI_H) CAI libraries and expressed them in Escherichia coli DH10B. They quantified mRFP expression using both flow cytometry and microplate reader measurements, normalizing fluorescence to account for variations in cell density. The full-length coding sequence (675 bp) for each variant was determined by Sanger sequencing. They applied quality control criteria to ensure data integrity, excluding samples with low-quality sequencing reads, amino acid mutations, mixed populations, or significant deviations between measurement methods. This curation process resulted in a high-quality dataset that provides a foundation for investigating the determinants of translation efficiency in *E. coli.* We accessed this dataset through the public repository as provided by the original authors and used it as the basis for our machine learning approach to predict protein production levels from mRNA sequence features.

For the SARS-Cov-2 vaccine degradation dataset, this dataset includes mRNA constructs encoding a multi-epitope vaccine (MEV) candidate based on SARS-CoV-2 antigens. The component of this dataset that we focus on in our experiments is the in-cell mRNA stability via time-course degradation experiments in HEK293T cells. This dataset, as described by Leppek et al. (2022), provides a resource for investigating the relationships between mRNA sequence, structure, stability, and expression efficiency in the context of SARS-CoV-2 vaccine design.

The Tc-riboswitch dataset (Groher et al., 2018) was developed to optimize the dynamic range (DR) and basal expression (BE) of tetracycline (Tc)-responsive synthetic riboswitches. These constructs consist of tandem Tc aptamers inserted into the $5'$ untranslated region (UTR) of a GFP reporter gene, regulating expression in response to Tc ligand binding. Using *Saccharomyces cerevisiae* RS453 as the host, GFP fluorescence was quantified with and without Tc induction via flow cytometry. Through machine learning-guided optimization, including random forest classifiers and convolutional neural networks, sequence and structural features influencing DR and BE were systematically explored by Groher et al. (2018). The curated dataset includes constructs with optimized biophysical properties, providing a foundation for understanding riboswitch function.

The MLOS dataset (Li et al., 2024) contains 164 mRNA candidates designed to encode the influenza hemagglutinin antigen, constructed with fixed untranslated regions and variable coding regions. These candidates were synthesized and transfected into cells to evaluate their performance. Additionally, the benchmarking dataset incorporated sequences from Sanofi encoding the hemagglutinin antigen for flu vaccines. Specifically, these mRNA sequences were evaluated for protein expression levels in HeLa cells.

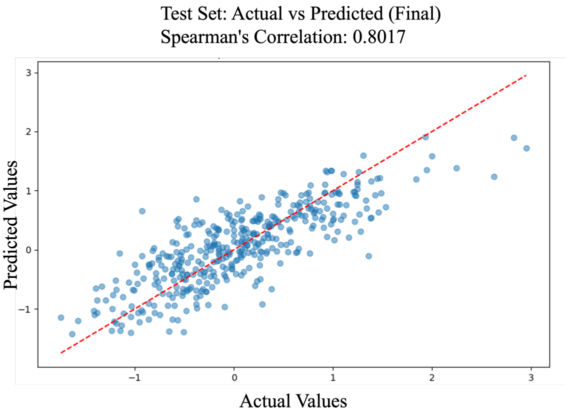

(a) Caduceus pretrained model feature effectiveness on SARS-CoV-2 vaccine degradation dataset.

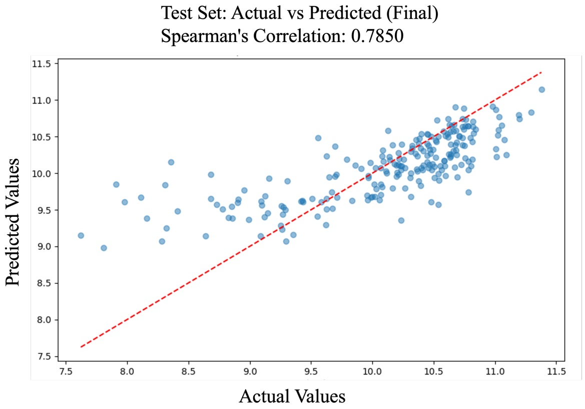

(b) Caduceus pretrained model feature effectiveness on mRFP expression dataset.

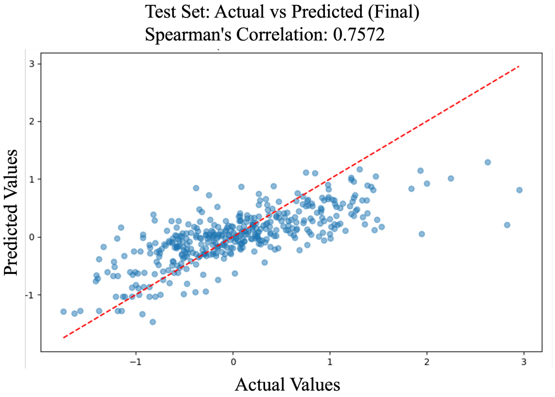

(c) HyenaDNA pretrained model feature effectiveness on SARS-CoV-2 vaccine degradation dataset.

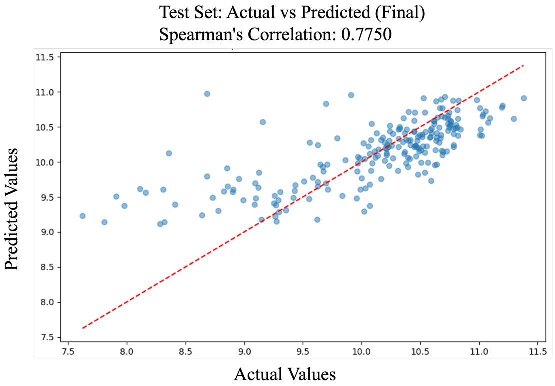

(d) HyenaDNA pretrained model feature effectiveness on mRFP expression dataset.

Figure 2 Evaluation of DNA pretrained model feature effectiveness on mRFP expression and SARS-CoV-2 vaccine degradation dataset using TextCNN.

A.4. Evaluation of DNA pretrained model feature effectiveness

We explore the ability of DNA-pretrained backbones, specifically Caduceus and HyenaDNA, to effectively generalize to mRNA-related tasks using a TextCNN framework. The tasks evaluated include predictions on the SARS-CoV-2 vaccine degradation dataset and the mRFP expression dataset. The scatter plots in Figure 2 show the alignment between actual and predicted values, with a trendline indicating overall correlation.

The SARS-CoV-2 vaccine degradation dataset serves as a proxy for evaluating the potential of DNA-pretrained features to capture complex biological dependencies related to RNA sequence stability and degradation. Both Caduceus and HyenaDNA demonstrate a clear trend of alignment between actual and predicted values, reflecting the potential of DNA-derived features to transfer effectively to mRNA stability prediction. While there are inherent challenges in modeling degradation, as indicated by a wider spread in predictions, the performance reflects the potential of pretrained DNA models to generalize beyond their training domain to tasks with overlapping biological mechanisms, such as RNA stability. The effectiveness of these features suggests that key structural and sequence-specific attributes learned from DNA datasets are applicable to mRNA-related degradation tasks.

The mRFP expression dataset focuses on the predictability of gene expression levels based on underlying sequence features. Both models achieve a closer alignment of predicted values to the actual values compared to the degradation dataset. This suggests that the DNA-pretrained features can be potentially effective at tasks involving expression prediction, where sequence features such as promoter regions, codon optimization, and untranslated regions are critical. The high clustering around the trendline demonstrates that these DNA backbones successfully capture sequence motifs and structural patterns that are transferable to mRNA-related tasks. This finding aligns with the hypothesis that DNA and RNA share significant overlapping biological motifs, enabling effective transfer learning.

A.5. Comparison with traditional feature-based approaches

To provide a more comprehensive evaluation of our approach against the broader landscape of RNA analysis methods (Li et al., 2024), Table 8 presents an expanded comparison that includes traditional feature-based methods not discussed in the main text. These methods represent baselines that have historically been used for genomic sequence analysis before the advent of deep learning approaches.

Table 8 Additional evaluation of computational complexity and Spearman's rank correlation metrics across RNA and DNA language models: measuring the impact of CodonMoE integration on model performance and parameter efficiency. Each data set is split into training, validation, and testing with a 0.7, 0.15, and 0.15 ratio, using the same split set as in the CodonBERT (Li et al., 2024). The metric is Spearman's rank correlation. **Size:** □ $<$ 10M, □□ $=$ 20–80M, □□□ $>$ 80M; **Complexity:** $O(L)$ - linear, $O(L \log L)$ - linearithmic, $O(L^2)$ - quadratic, $O(N)$ - linear (L = sequence length, N = vocabulary size);

**Model**	**Type**	**Params**	**Size**	**Complexity**	**Vaccine**	**mRFP**	**Tc-ribo.**
Traditional Feature-based Methods							
TF-IDF	Codon	–	–	$O(N)$	0.69	0.57	0.49
Plain TextCNN	Codon	0.15M	□	$O(L)$	0.80	0.78	0.43
Codon2vec+TextCNN	Codon	0.15M	□	$O(L)$	0.70	0.77	0.56
DNA Foundation Models Enhanced with CodonMoE							
HyenaDNA + CodonMoE-pro (ours)	Codon	7.50M	□	$O(L \log L)$	**0.84**	**0.88**	**0.60**

The traditional feature-based approaches include TF-IDF (Term Frequency-Inverse Document Frequency), which treats codons as vocabulary terms and measures their frequency distribution across sequences. Despite its simplicity and lack of trainable parameters, TF-IDF achieves reasonable performance on stability prediction tasks on the vaccine degradation dataset.

Plain TextCNN applies convolutional neural networks directly to codon sequences without extensive pre-training, using shallow architectures with approximately 0.15M parameters. This lightweight approach performs surprisingly well on certain tasks, achieving competitive performance on vaccine degradation prediction.

Codon2vec+TextCNN employs pre-trained codon embeddings (similar to word2vec in natural language processing) before applying convolutional layers, providing stronger semantic representations of codons with minimal parameter overhead. This method shows balanced performance across tasks, particularly on the Tc-riboswitch dataset where it matches several larger models.

The more comprehensive comparison on three datasets demonstrates that while our CodonMoE-enhanced DNA models achieve state-of-the-art performance across most tasks, certain traditional approaches remain competitive for specific applications despite their significantly lower computational requirements. This suggests that task-specific inductive biases can sometimes compensate for model scale, particularly when biological mechanisms align well with the model architecture.

A.6. Additional introduction of genomic backbones

RNABERT (Akiyama and Sakakibara, 2022) and RNA-FM (Chen et al., 2022) represent the first generation of nucleotide-level RNA language models that learn sequence and structural representations from large non-coding RNA corpora. RNABERT, trained on 76,237 ncRNA sequences from RNAcentral using masked language modeling and structural alignment learning, integrates local nucleotide context with secondary structure information through a six-layer Transformer. RNA-FM expands this paradigm with a 12-layer bidirectional Transformer trained on 23 million ncRNA sequences, capturing long-range dependencies and evolutionary signals to produce generalizable embeddings for RNA structure and function prediction.

Building on these nucleotide-level foundations, CodonBERT (Li et al., 2024) introduces codon-based modeling for mRNA, employing a 12-layer, 768-dimensional BERT architecture trained on 10 million coding sequences across 13 evolutionary groups. By combining Masked Language Modeling and Homologous Sequence Prediction, CodonBERT captures translation-level and evolutionary constraints critical for mRNA optimization. Complementing these RNA models, GPN-MSA (Benegas et al., 2023) integrates evolutionary conservation from multiple-sequence alignments of 100 vertebrates into a RoFormer (Su et al., 2024) framework for variant effect prediction, emphasizing conserved regions through phastCons and phyloP scores.

More recently, HyenaDNA (Nguyen et al., 2024b) and Caduceus (Schiff et al., 2024) advance genomic modeling by addressing long-range dependencies and strand symmetry: HyenaDNA leverages the Hyena operator to process million-token contexts with sub-quadratic scaling and in-context adaptability, while Caduceus introduces RC-equivariant MambaDNA blocks for bidirectional, strand-invariant learning, achieving state-of-the-art performance on diverse regulatory and variant effect prediction tasks.

A.7. Biological interpretability of codon experts

To characterize the biological specializations learned by CodonMoE series, we analyze the MLOS dataset as our primary interpretability testbed. All 164 mRNA candidates in this dataset share identical untranslated regions and differ only in their coding sequences, making any differences in model behavior attributable entirely to codon usage.

A.7.1. Expert routing pattern

We extracted the soft gating weights from the Adaptive Mixture of Codon Experts layer for all 64 codons across sequences in the held-out test and validation sets (Figure 3). The four experts exhibit distinct, non-redundant specializations. Expert 4 preferentially activates for translationally optimal codons, its top-5 codons (CAG, CUG, CCG, CGG, GAG) are all NNG codons representing very frequently used synonymous codons in the human genome. Expert 2 specializes in stop codons and rare codons, with its top-3 codons being all three stop codons (UAA, UAG, UGA), indicating that the model has learned to recognize translational termination signals without explicit supervision. Expert 3 serves as a general-purpose expert with relatively uniform activation, while Expert 1 captures AU-rich codon patterns (UCU, UCA, ACA).

Importantly, synonymous codons encoding the same amino acid show markedly different routing patterns (Figure 4). For example, among the six Leucine codons, CUG is routed primarily to Expert 4 (gate weight 0.52), while UUA is routed to Experts 2 and 3 (gate weights 0.15 and 0.43, respectively), confirming that the model captures codon-level rather than amino acid-level features.

A.7.2. Correlation with known codon properties

We computed Pearson correlations between expert gate weights and known codon properties across all 64 codons (Figure 5). Expert 4 shows significant positive correlation with GC content ($r = 0.52$, $p < 0.001$) and human codon usage frequency ($r = 0.45$, $p < 0.001$). Expert 2 exhibits significant negative correlations with the same metrics (GC content: $r = -0.55$, $p < 0.001$; codon frequency: $r = -0.49$, $p < 0.001$; GC3 wobble position: $r = -0.50$, $p < 0.001$). This complementary expert structure, one expert specializing in optimal, GC-rich codons and another in rare, AU-rich codons, recapitulates the well-established biological relationship between codon optimality and translational efficiency. Scatter plots (Figure 6) confirm these correlations are robust and not driven by individual outlier codons.

A.7.3. Convolutional filter analysis

We analyzed the learned convolutional filters ($k = 3, 4, 5$ codons) in CodonMoE-pro by identifying codon patterns that maximally activate each filter (Figures 7 and 8). Filter importance follows a long-tailed distribution, with a small number of filters contributing disproportionately to predictions. The most important filters preferentially activate for clusters of high-frequency optimal codons (CUG, AUC, AAC, GAA), consistent with the expert routing patterns. This suggests that the MoE and convolution components work synergistically: the MoE layer identifies codon-level properties at individual positions, while the convolution captures local clustering patterns of optimal or non-optimal codons across neighboring positions.

A.8. Expert number ablation study

Table 9 Effect of expert number K on mRFP expression and vaccine degradation (Spearman's ρ, test set). Base model: HyenaDNA + CodonMoE-pro. $K = 4$ achieves the best performance on codon-sensitive expression prediction; vaccine degradation is robust across K, consistent with the dominance of the convolutional component for stability tasks.

K	mRFP	Vaccine
1	0.83	**0.85**
2	0.85	0.84
4	**0.88**	0.84
6	0.84	0.83
8	0.83	0.84

On mRFP expression, where codon usage bias directly influences translational efficiency, $K = 4$ substantially outperforms all other configurations. The drop at $K = 1$ ($\Delta\rho = -0.05$) confirms that multiple specialized experts are essential for capturing codon-level properties. Increasing beyond $K = 4$ slightly degrades performance, suggesting overfitting from over-partitioning the codon space with limited training data.

On vaccine degradation, performance remains stable across K values ($\rho = 0.83$–0.85), indicating that the codon reshaping and convolutional components, present at all K values, drive the improvement over raw HyenaDNA, while expert number has minimal impact. This task-dependent sensitivity is biologically interpretable: expression prediction relies on fine-grained codon optimality distinctions that benefit from specialized experts, whereas stability prediction depends more on local sequence motifs captured by the convolutional module.

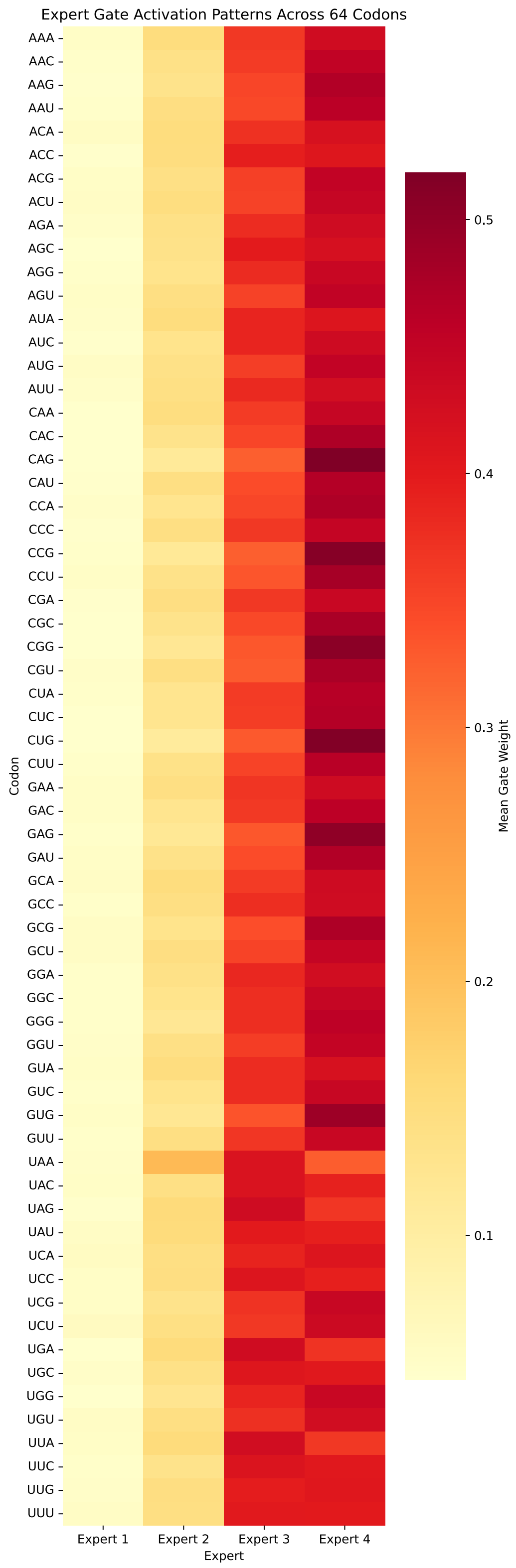

Figure 3 Expert gating weights across 64 codons.

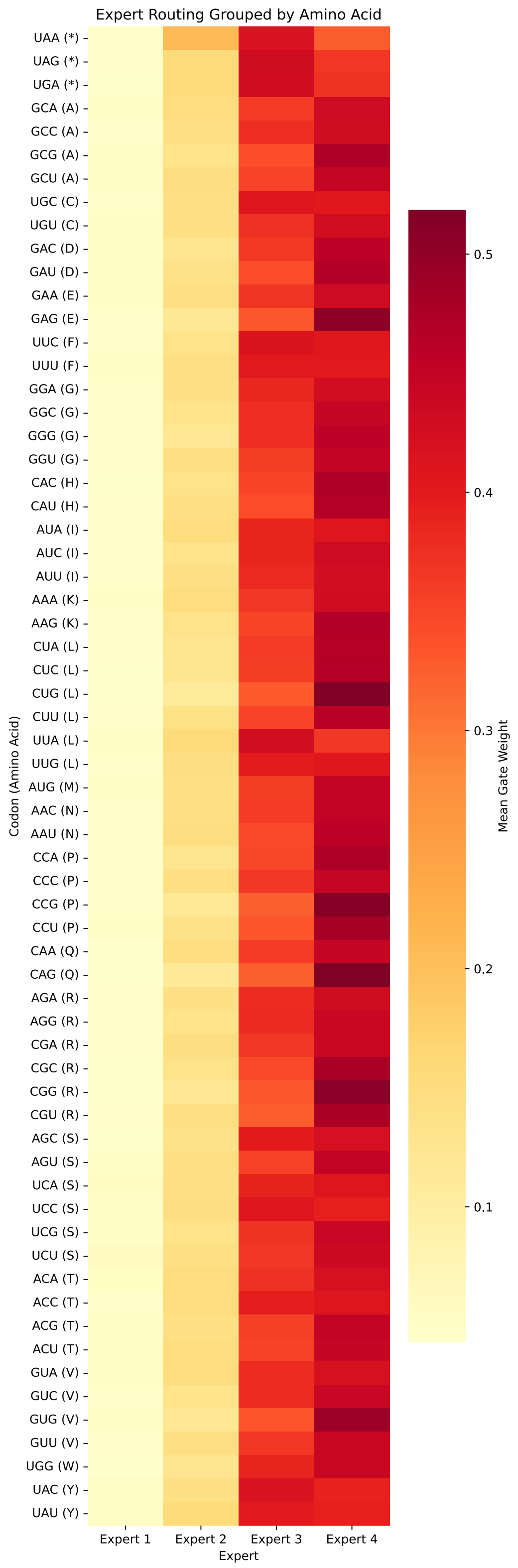

Figure 4 Expert gating weights grouped by amino acid. Synonymous codons encoding the same amino acid exhibit distinct routing patterns, confirming codon-level specialization.

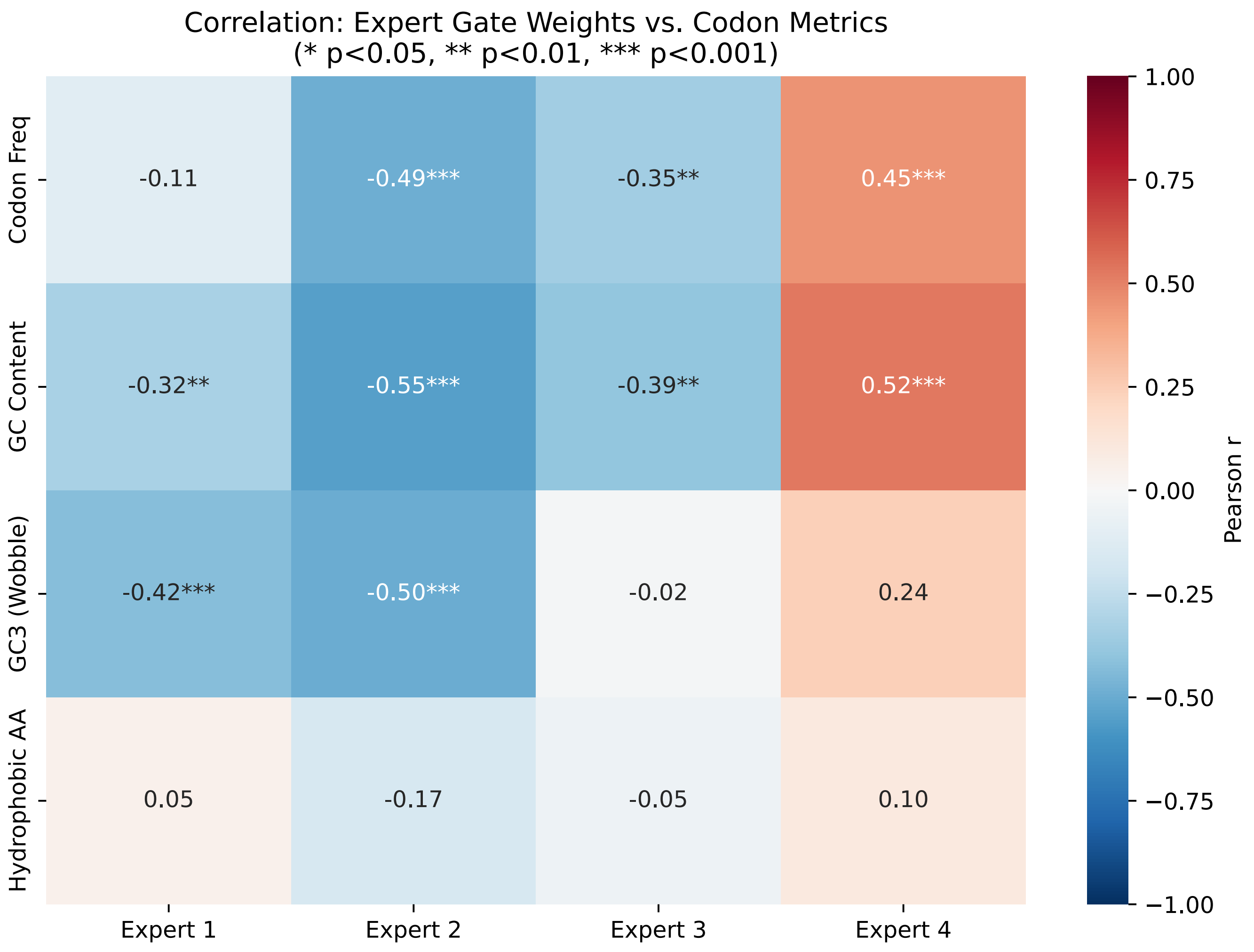

Figure 5 Pearson correlation heatmap between expert gate weights and four codon properties (codon frequency, GC content, GC3 wobble position, hydrophobic amino acid). $^{*}p < 0.05$, $^{**}p < 0.01$, $^{***}p < 0.001$.

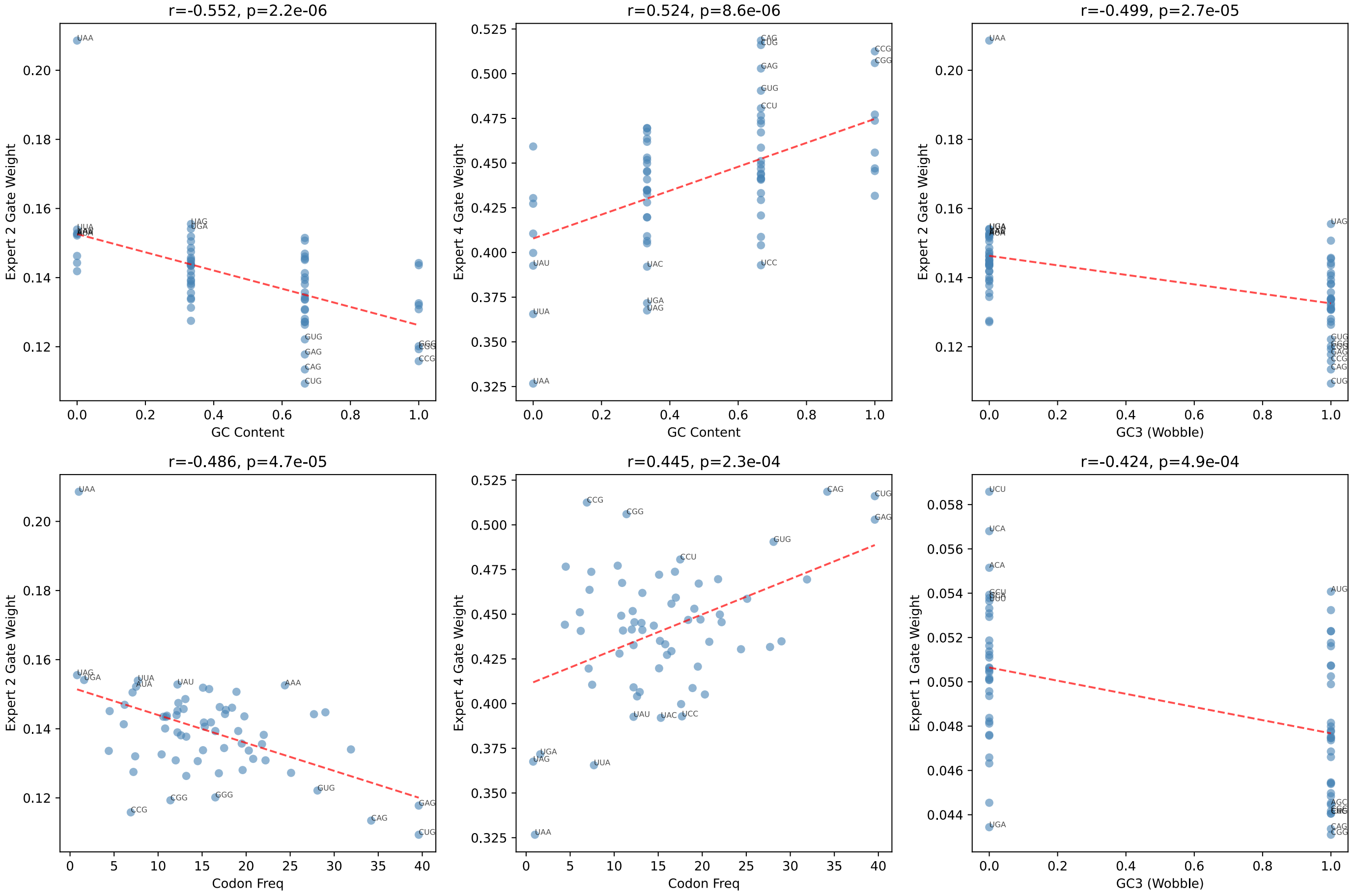

Figure 6 Scatter plots for the six most significant expert–metric correlations, confirming that correlations are robust and not driven by outlier codons.

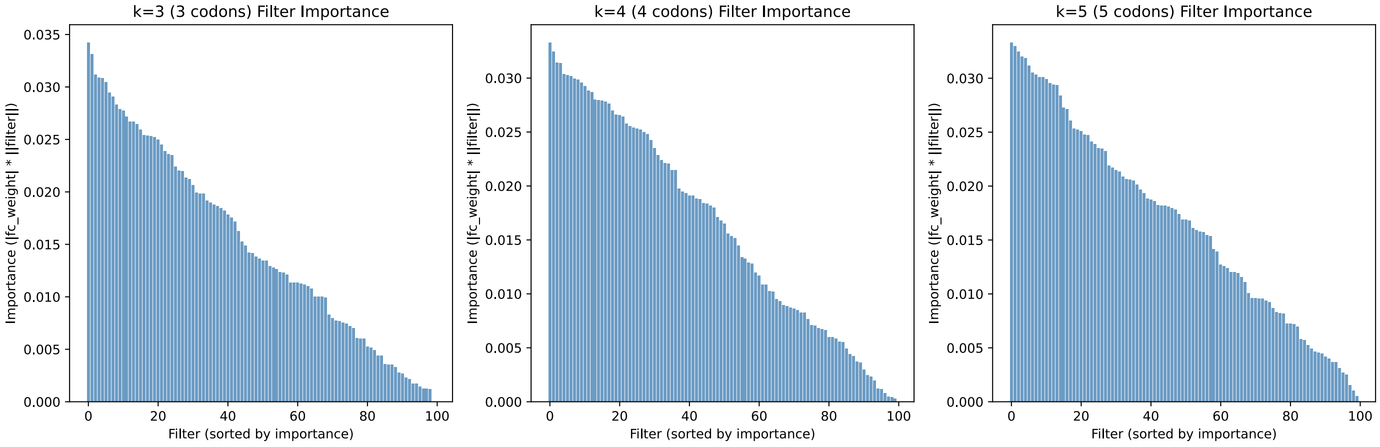

Figure 7 Filter importance distributions (sorted by decreasing importance) for $k = 3, 4, 5$ codon convolution kernels.

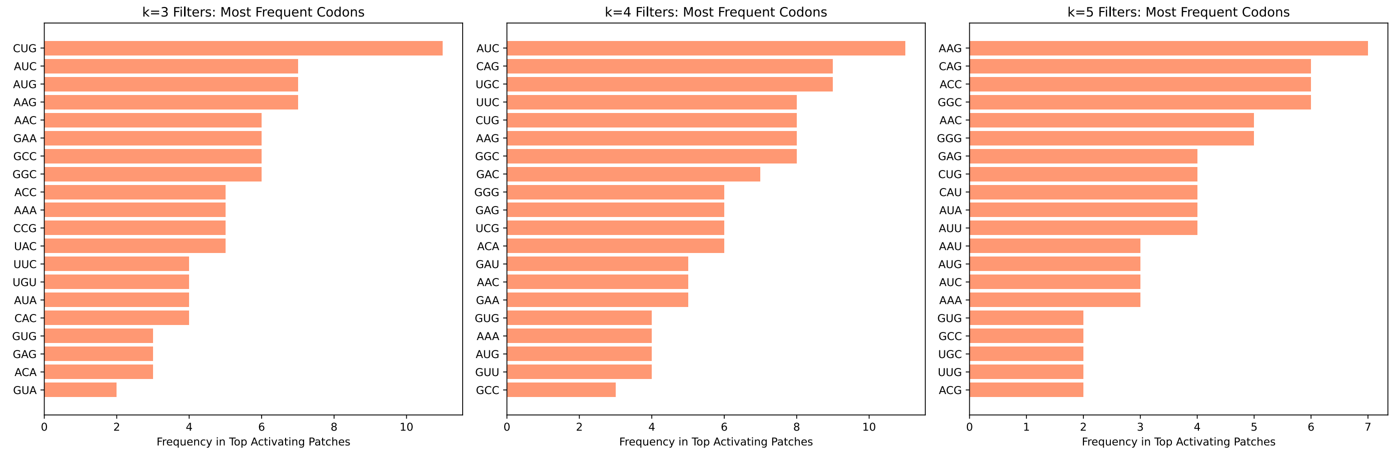

Figure 8 Most frequently occurring codons in the top-activating patches for each kernel size ($k = 3, 4, 5$).
